# The co‐chaperone p23 promotes prostate cancer motility and metastasis

**DOI:** 10.1016/j.molonc.2014.08.014

**Published:** 2014-09-06

**Authors:** Laia Querol Cano, Derek N. Lavery, Soraya Sin, Emma Spanjaard, Greg N. Brooke, Jessica D. Tilman, Ahmed Abroaf, Luke Gaughan, Craig N. Robson, Rakesh Heer, Francesco Mauri, Johan de Rooij, Keltouma Driouch, Charlotte L. Bevan

**Affiliations:** ^1^ Androgen Signalling Laboratory, Imperial Centre for Translational & Experimental Medicine, Imperial College London, London W12 0NN, UK; ^2^ Genetics Laboratory, Department of Tumor Biology, Institut Curie, Paris 75248, France; ^3^ Hubrecht Institute for Developmental Biology and Stem Cell Research and University Medical Centre Utrecht, Uppsalalaan 8, 3584 CT Utrecht, The Netherlands; ^4^ Northern Institute for Cancer Research, Newcastle University, Newcastle Upon Tyne NE2 4HH, UK; ^5^ Department of Histopathology, Imperial Healthcare NHS Trust, Hammersmith Hospital, London W12 0NN, UK

**Keywords:** p23, Androgen receptor, Chaperone, Prostate cancer, Metastasis, Heat shock protein 90

## Abstract

Prostate cancer is an androgen receptor (AR)‐dependent malignancy at initiation and progression, therefore hormone therapy is the primary line of systemic treatment. Despite initial disease regression, tumours inevitably recur and progress to an advanced castration‐resistant state a major feature of which is metastasis to the bone. Up‐regulation of AR cofactors and chaperones that overcome low hormone conditions to maintain basal AR activity has been postulated as a mechanism of therapy relapse.

p23, an essential component of the apo‐AR complex, acts also after ligand binding to increase AR transcriptional activity and target gene expression, partly by increasing chromatin‐loaded holo‐receptor‐complexes. Immunohistochemical studies have demonstrated increased p23 expression in advanced prostate cancer. Here, we further characterise p23 roles in AR signalling and show that it modulates cytosolic AR levels in the absence of hormone, confirming a chaperoning function in the aporeceptor complex and suggesting p23 upregulates AR signalling at multiple stages. Moreover, p23 protein levels significantly increased upon treatment with not only androgen but also clinically relevant anti‐androgens. This was in contrast to the HSP90 inhibitor 17‐AAG, which did not modulate expression of the cochaperone – important given the HSP90‐independent roles we and others have previously described for p23.

Further, we demonstrate p23 is implicated in prostate cancer cell motility and in acquisition of invasiveness capacity through the expression of specific genes known to participate in cancer progression. This may drive metastatic processes in vivo since analysis of prostate tumour biopsies revealed that high nuclear p23 significantly correlated with shorter survival times and with development of metastases in patients with lower grade tumours. We propose that increased p23 expression may allow cells to acquire a more aggressive phenotype, contributing to disease progression, and that p23 is a plausible secondary target in combination with HSP90 inhibition as a potential therapy for advanced prostate cancer.

## Highlights

- We report a novel function for p23 in prostate cancer progression.
- p23 protein levels increase upon treatment with androgens and anti‐androgens.
- p23 promotes prostate cancer cell motility and acquisition of invasiveness.
- High nuclear p23 significantly correlates with shorter survival times in patients.
- p23 may reprogram gene expression profile into a more metastatic pattern.

## 1 Introduction

The molecular chaperone p23 is ubiquitously expressed and highly conserved from yeast to humans (Felts and Toft, 2003; Garcia‐Ranea et al., 2002). It was first characterised as a component of the progesterone receptor (PR) complex but is an intrinsic component of several other steroid receptor (SR) hetero‐complexes, exerting distinct effects on different receptors (Freeman and Yamamoto, 2002; Hutchison et al., 1995; Nair et al., 1996; Reebye et al., 2012). For instance, p23 has been shown to decrease glucocorticoid receptor (GR) activity by disassembling receptor hetero‐complexes from chromatin, but to increase activity of the androgen and oestrogen receptors (AR and ER) (Freeman and Yamamoto, 2002; Knoblauch and Garabedian, 1999; Oxelmark et al., 2006; Reebye et al., 2012). Encoded by the *PTGES3* gene, p23 is an acidic 160 amino acid protein that can be divided into two domains, an amino‐terminal region containing an HSP90 binding site (residues 86–108) and an unstructured carboxyl terminal domain (Ali et al., 2006; Martinez‐Yamout et al., 2006; Weikl et al., 1999; Zhu and Tytgat, 2004). p23 is the smallest component of the HSP90 chaperone machinery, with a molecular weight of 23,000 Da, and is mainly known for binding the ATP‐bound form of HSP90, inhibiting its intrinsic hydrolytic activity and stabilising several HSP90‐substrate complexes including Fes tyrosine kinase, transcription factors such as HSF1, telomerase and the reverse transcriptase enzyme (Holt et al., 1999; Hu et al., 2002; Nair et al., 1996). Further highlighting the importance of p23 in cellular function and development, a knockout mouse model exhibited prenatal or perinatal lethality and p23 has also been shown to possess prostaglandin E2 synthase activity, although the significance of this remains unclear since p23 knockout mice do not exhibit impaired prostaglandin enzymatic activity (Felts and Toft, 2003; Grad et al., 2006; Lovgren et al., 2007; Tanioka et al., 2000).

Although p23 has historically been studied as an HSP90 co‐chaperone, a number of studies published over recent years suggest it also exerts some of its functions in an HSP90‐independent manner. Several groups have demonstrated that p23 can continue acting on proteins that have been released from HSP90 and p23 has also been shown to possess a passive, ATP‐independent chaperoning activity in the C‐terminus and suppress the aggregation of denatured proteins (Bose et al., 1996, 2000, 1996, 2002, 2012). Our laboratory has shown that the interaction between p23 and AR is at least partially HSP90‐independent and that a mutant form of p23 unable to bind HSP90 significantly enhanced AR transcriptional activity to a similar extent as observed for wild type p23 (Reebye et al., 2012).^3^

There is considerable evidence that p23 could be implicated in cancer processes as it has been shown to be up‐regulated in several tumour types, including lung, prostate and breast as well as acute lymphoblastic leukaemia (Elmore et al., 2008; Krebs et al., 2002; Li et al., 2009; Liu et al., 2012; Mollerup et al., 2003; Oxelmark et al., 2006; Reebye et al., 2012). The role of p23 in breast cancer has been extensively studied by the Garabedian laboratory, who showed p23 enhances cell motility and that higher levels correlate with poor prognosis and a reduction in disease‐free survival time in breast cancer patients (Oxelmark et al., 2006, 2012, 2010). We have previously shown nuclear p23 to enhance AR activity and binding to chromatin, critical steps for AR signalling and prostate cancer development, and to be increased with tumour grade (Reebye et al., 2012). Similarly to what has been described in breast cancer, here we show that p23 also affects prostate cancer cell migration and invasion properties without affecting cell growth. Moreover, p23 may mediate these effects by selectively modulating the expression of genes previously involved in metastatic processes. Supporting clinical significance, nuclear p23 correlates with a decrease in survival in patients with Gleason score ≤7 and with an increase in metastatic progression.

Prostate cancer is, in its early stages, an AR‐dependent malignancy and non‐curative treatments, such as anti‐androgens, are designed to inhibit AR signalling. Despite high initial success, therapeutic failure very frequently occurs and the malignancy progresses to a hormone‐refractory stage for which no effective treatment has been yet developed. HSP90 inhibitors such as geldanamycin and synthetic derivatives e.g. 17‐AAG are now in clinical trials for prostate cancer. These drugs inhibit HSP90 largely by preventing its interaction with p23. However, the yeast p23 homologue, Sba1, was shown to protect cells against the effects of HSP90 inhibitors such as geldanamycin or radicicol (Forafonov et al., 2008) and here we show that both androgen and antiandrogens can increase p23 protein levels. In combination with our observations regarding the positive effects of p23 upon AR stabilisation and signalling, and also on cell aggressiveness and motility, this leads us to postulate that inhibiting p23 in combination with HSP90 could be an effective therapeutic combination against prostate cancer.

## 2 Experimental procedures

### 2.1 Cell culture

LNCaP, C4‐2 and PC‐3 cells were cultured in RPMI‐1640 (Sigma) supplemented with 100 U/ml penicillin, 0.1 mg/ml streptomycin, 2 mmol/L glutamine (Sigma) and 10% fetal bovine serum (Labtech International, Ringmer, East Sussex, UK). LNCaP‐TR2‐MAR4‐p23‐V5 cells (referred to as LNCaP‐p23 throughout) (Reebye et al., 2012) were grown as above but using certified tetracycline‐free‐medium (Firstlink UK, Sheffield, UK).

### 2.2 Time courses

LNCaP cells were cultured in 6‐well plates at a density of 4 × 10^5^ cells/well in hormone‐depleted medium, comprising phenol red‐free RPMI (Sigma) supplemented as above but with 5% charcoal‐stripped fetal bovine serum (Labtech International), for 72 h prior to treatment with 10 nM mibolerone (Mib) (PerkinElmer Life Sciences, Wellesley, MA), 1 μM bicalutamide (Bic) (Sigma, Chemical Co., St. Louis, MO) or 10 nM 17‐allylamino‐17‐demethoxygeldanamycin (17‐AAG) (Sigma). Cells were harvested for protein extraction and protein expression assessed by Western blotting using 10 μg of total lysate.

### 2.3 Immunoblotting

Equal amounts of total protein were separated by SDS‐PAGE, transferred onto PVDF membrane (Millipore, Watford, UK) and probed using primary antibodies: mouse anti‐p23 (1:3000, ab2814, Abcam), rabbit anti‐AR (AR441, 1:1000, Dako), rabbit anti‐HSP90 (sc7977, 1:500, SantaCruz), mouse anti‐β‐actin (ab8226, 1:8000, Abcam), mouse anti‐αtubulin (DM1A, 1:1000, Sigma) and mouse anti‐vinculin (V9131, 1:1000, Sigma). Proteins were detected using horseradish peroxidise‐conjugated anti‐mouse or anti‐rabbit secondary antibodies (1:2000, DAKO) and visualised using the ECL system (GE Healthcare).

### 2.4 RNA preparation and quantitative real‐time PCR

Total RNA was extracted using the RNeasy extraction kit (QIAGEN, Chatsworth, CA) and 500 ng RNA reverse transcribed using the reverse transcriptase kit (PrimerDesign). LAMB1, KYNU, CTSD and PMP22 expression was assessed by quantitative real‐time PCR using Fast Reaction SYBR‐Green mixture (Applied Biosystems). Data were normalized to GAPDH expression. Primer sequences are shown in Supplemental Table S1.

### 2.5 Transient knockdowns

LNCaP cells were grown to 70% confluence in 6‐well plates, transferred to starvation medium for 24 h before transfection with 100 nM/well p23‐targeting small interfering RNA(siRNA) pool (Dharmafect L‐004496–00; Dharmacon, Lafayette,CO) or scrambled negative control using 2 μl of Dharma‐FECT‐2 transfection reagent (Dharmacon). Cells were harvested for protein extraction 72, 96 and 120 h after transfection and protein expression was assessed by Western blotting using10 μg of protein lysate. For AR knockdown experiments, LNCaP‐AR‐shRNA or LNCaP‐AR‐scrambled inducible cell lines (kind gift from Paul Rennie, University of British Columbia, Vancouver, Canada (Cheng et al., 2006)) were treated with 2 μM doxycycline (DOX) for 72, 96 and 120 h prior to being harvested for protein extraction and Western blotting performed as above.

### 2.6 Transwell assays

Migration assays were performed in triplicate using Companion 24‐well plates and transwell chambers (both BD Falcon) with 8 μm pore size membranes previously coated with 100 μl of collagen diluted to 25 μg/ml using serum free medium (Invitrogen) for 2 h at room temperature. LNCaP‐p23 cells pre‐treated with 100 nM DOX or vehicle control for 48 h, or LNCaP cells previously transfected with specific siRNA against p23 or non‐targeting siRNA as negative control, were seeded at a density of 1.5 × 10^4^ cells/transwell in serum free medium into the upper chamber of the transwell. The bottom chamber was filled with 650 μl of RPMI‐1640 medium containing 10% FCS and left at 37 °C for 24 h. Invasion assays were performed as above but the top chamber was also pre‐coated with 4 μg/cm^2^ of matrigel (BD biosciences) for 3 h prior to cell seeding. The contents of the upper chamber were then scraped out and the filter fixed by incubation with 4% PFA for 20 min at room temperature, stained with crystal violet and washed. Migration and invasion were quantified using ImageJ software and results normalised to the corresponding cell density.

### 2.7 Wound healing assays

LNCaP cells or PC3 cells were transfected with either siRNA against p23 or non‐targeting siRNA as negative control for 72 h prior to being seeded to confluence in 6‐well plates in full medium. Monolayers were scratched with a pipette tip and washed with PBS twice to remove cell debris. Cells were maintained in hormone‐depleted medium and imaged by contrast microscopy (Eclipse, TS100, Nikon). Images were captured at the time and 24, 36 and 48 h after scratching, or every 30 min up to 12 h for PC3 cells.

### 2.8 Proliferation assays

LNCaP cells previously transfected with siRNA p23 or a non‐targeting control, and LNCaP‐p23 cells treated with 100 nM DOX to enhance p23 expression or vehicle control, were seeded in 96‐well plates at a density of 1 × 10^4^ cells/well in full medium. Cell viability was assessed by incubation with 20 μl of the MTS reagent (Promega) for 2 h at 37 °C following manufacturer's instructions. Crystal violet assays were also performed and for this LNCaP cells were seeded in 96 well‐plates at a density of 2000 cells/well in full medium. Twenty‐four hours after seeding, cells were treated with 0.1, 0.5, 2.5, 5 or 10 μM 17‐AAG or DMSO as vehicle control for 16, 24, 48 and 72 h. After this time cells were fixed with 4% PFA, washed twice in PBS and stained with 0.8% crystal violet for 30 min at room temperature. Cells were then washed four times with water and left to dry for at least 24 h. Crystal violet was resolubilised in 100 μl 10% acetic acid and absorbance read at 595 nm.

### 2.9 Tissue microarray and immunohistochemistry

Immunohistochemistry was performed using 3 tissue microarrays (TMAs) of benign and malignant prostate biopsies derived from transrectal biopsy, transurethral resection, and radical prostatectomy as previously described (Brooke et al., 2011; Gaughan et al., 2011). All materials were used in accordance with approval granted by the Northumberland, Tyne and Wear NHS Strategic Health Authority Research Ethics Committee (reference 2003/11; The Freeman Hospital). The final study included 321 cancer biopsies and 69 benign biopsies. Antigen retrieval was achieved by immersion in 10 mmol/L citric acid buffer (pH 6.0), followed by microwaving for 15 min (at 1000 W) in a pressure cooker. Sections were immunostained with a mouse monoclonal antibody against p23 (Abcam) on a DAKO autostainer using Vectastain ABC kits (Vector Labs), according to the manufacturer's protocol. Sections known to stain positively were included in each batch, and negative controls were prepared by replacing the primary antibody with TBS buffer. p23 expression was scored blindly for epithelial nuclear intensity of staining and number of epithelial nuclei positive per field, in each biopsy core. Slides were scanned using a Scanscope GL scanner (Aperio) and analysed using SpectrumTM software (Aperio). For statistical analysis, samples were split into low or high intensity/number of positive nuclei (low = 0, 1 and high = 2, 3).

## 3 Results

### 3.1 Altering p23 levels affects AR stabilisation in the absence of ligand

**Figure 1:**
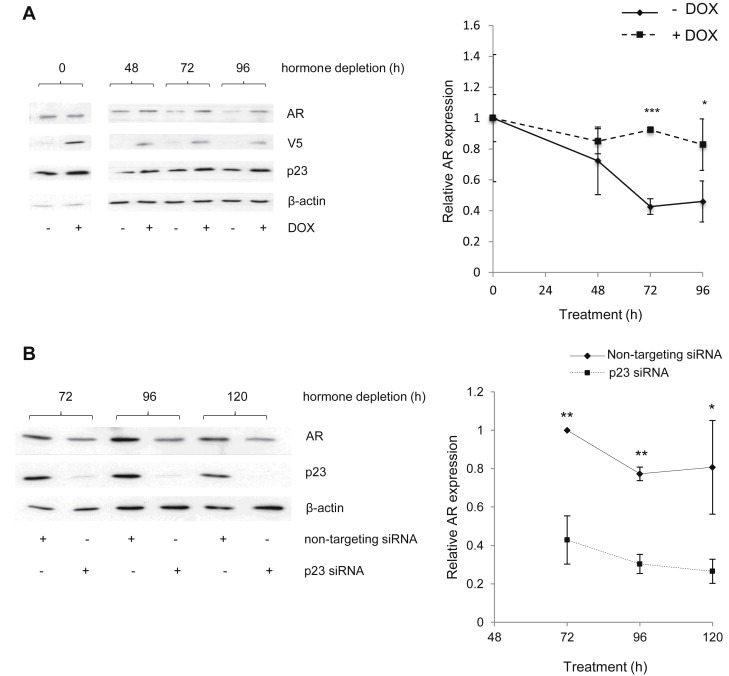
p23 regulates AR expression in the absence of hormone. (A) LNCaP‐p23 cells were treated with 100 nM doxycycline (DOX) for 48 h followed by a 48 h hormone‐depletion also in the presence of DOX. Cell lysates were collected at the indicated time points, harvested, resolved by Western blotting and transferred to PVDF membranes, which were probed using specific antibodies against AR, p23 and β–actin. Densitometry was performed on Western Blotting results normalising against signal obtained for β–actin. (B) LNCaP cells were transfected with specific siRNA against p23 collected at the indicated time points, harvested and resolved by Western Blotting. Gels were transferred onto PVDF membranes and probed with specific antibodies against p23, AR and β‐actin. Densitometry performed on Western results as above. Results presented are the mean ± 1 STDEV of three independent experiments. *p value ≤0.05 **p value ≤0.005 ***p value ≤0.0005 (Student's t test).

We have previously shown that increasing levels of p23 by ectopic expression significantly enhances AR transcriptional activity and, conversely, decreasing p23 levels using siRNA decreases it (Reebye et al., 2012). To further clarify the roles p23 may play within the AR signalling pathway, the effects of p23 over‐expression or knockdown upon AR protein levels were analysed under hormone‐depleted conditions. For this purpose we ectopically expressed V5‐tagged p23 using the LNCaP‐p23 cell line by incubating with 100 nM doxycycline (DOX) for 48 h in full media, to induce ectopic p23 expression, prior to culturing cells under starvation conditions for the specified times (Figure 1A). As shown, in the absence of DOX, AR levels decreased due to the hormone‐depleted conditions in which cells were cultured but increasing p23 abrogated this effect and levels of AR remained stable. This stabilisation was only evident under hormone‐depleted conditions, since prior to starvation at the 0 h time point no differences were detected between untreated and DOX‐treated samples.

The converse experiment was also performed and parental LNCaP cells were transfected with siRNA against p23, or a non‐targeting control siRNA, and cells harvested at the specified time points. In agreement with observations above, decreasing p23 significantly diminished AR protein levels for all time points analysed (Figure 1B). Together, this suggests p23 plays a key role in stabilising AR.

### 3.2 Effects of androgen upon p23 protein levels

**Figure 2:**
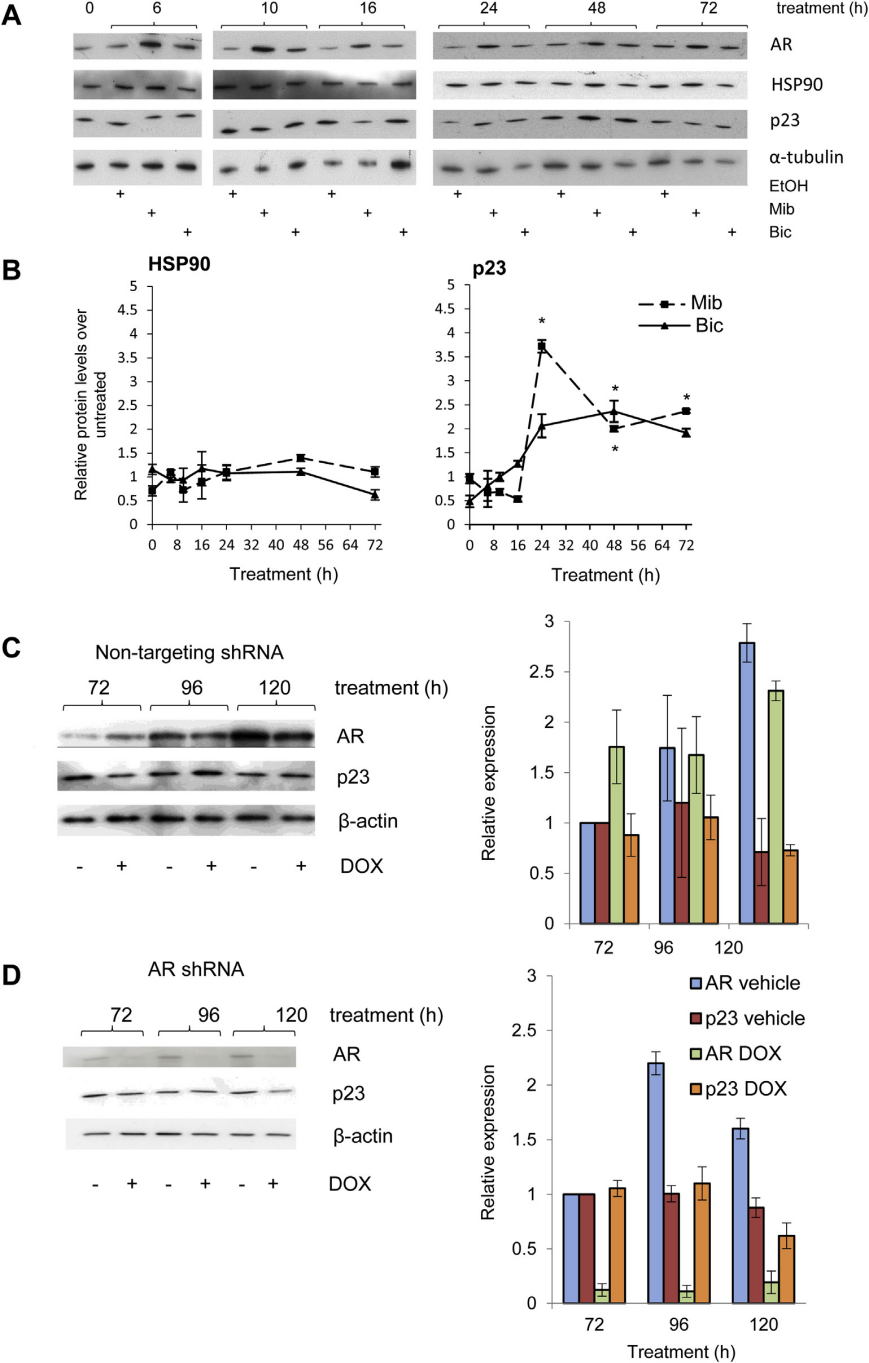
p23 regulation. (A) LNCaP cells were stripped for 72 h prior to treatment with 10 nM mibolerone (Mib) or 1 μM bicalutamide (Bic) for the indicated time points. Cells were harvested and lysates resolved by Western blotting, transferred onto PVDF and membranes probed with specific antibodies against HSP90, p23 and α–tubulin. (B) Graphs for HSP90 and p23 represent densitometry performed on Western‐blot images for each of the treatments and normalised against the signal obtained for α‐tubulin. Mib and Bic treated samples were then normalised against the corresponding untreated control for each time point, hence vehicle treated results have been removed from graphs for clarity. Results presented are the mean ± 1 standard error of three experiments. LNCaP cells stably transfected with scrambled (C) or shRNA against AR (D) were treated with 2 μM doxycycline (DOX) for 48 h. Cells were lysed at the indicated time points and proteins resolved by SDS‐PAGE and probed with antibodies against AR, p23 and β‐actin. Figures are a representative result of four experimental replicates. Graphs for either the scrambled (C) or shRNA transfected line (D) represent densitometry performed on western results, normalising against signal obtained for β‐actin. The shading is the same for both C and D. Results presented are the mean ± 1 standard error of four independent experiments. * = p ≤ 0.05; ** = p ≤ 0.005 (Student's test).

Very little is known regarding the mechanisms regulating p23 expression despite the essential roles p23 plays in cell function. We further examined the regulation of p23 in the context of AR signalling and prostate cancer by treating LNCaP cells with 10 nM of the synthetic androgen mibolerone (Mib) (Figure 2A). From analyses of cell lysates we observed that whereas addition of Mib had no effect upon HSP90, it caused a significant increase in p23 levels at and beyond 24 h treatment, suggesting p23 protein could be androgen‐stabilised or induced (Figure 2B). To further clarify the involvement of androgens and their receptor in p23 regulation, the LNCaP‐AR‐shRNA cell line, in which AR knockdown is induced upon DOX treatment (Cheng et al., 2006), was used to study the effects of AR on p23 expression. AR knockdown had no effect upon p23 protein levels since no differences were observed in DOX‐treated cells compared to those treated with vehicle (Figure 2D) – as a control, no significant changes in either protein were observed in the control scrambled shRNA‐transfected cell line (Figure 2C).

**Figure 3:**
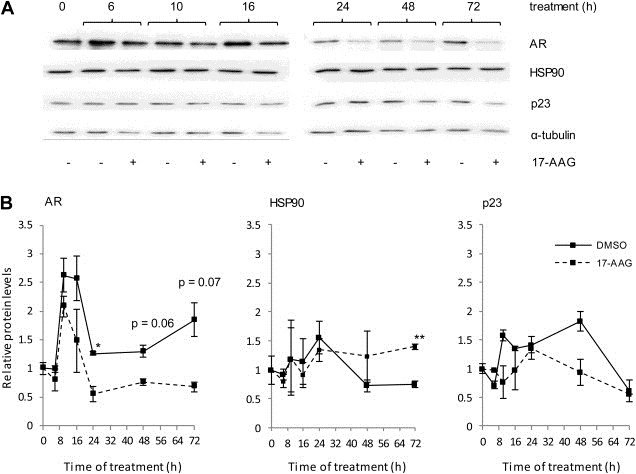
p23, HSP90 and AR expression in response to 17‐AAG. (A) LNCaP cells were incubated in hormone‐depleted medium for 72 h prior to treatment with 10 nM of the HSP90 inhibitor 17‐AAG. Cells were harvested and lysates resolved by Western blotting, transferred onto PVDF and membranes probed with specific antibodies against AR, HSP90, p23 and α–tubulin. (B) Graphs for HSP90 and p23 represent densitometry performed on Western‐blot images for each of the treatments and normalised against the signal obtained for α‐tubulin. Results presented are the mean ± 1 standard error of three independent experiments. * = p ≤ 0.05; ** = p ≤ 0.005 (Student's test).

We also investigated the effects on p23 protein levels after inhibiting either AR (using the therapeutic anti‐androgen bicalutamide (Bic)) or the HSP90‐p23 interaction (using the HSP90 inhibitor 17‐AAG, currently in phase II clinical trials (Neckers and Workman, 2012)). LNCaP cells were seeded in starvation medium prior to treatment with 1 μM of Bic (Figure 2A,B) or 10 nM of 17‐AAG (Figure 3A,B). As seen for Mib, addition of Bic did not have an effect on HSP90 endogenous levels, which remained similar to untreated cells (Figure 2B). p23 endogenous levels increased over vehicle‐treated up to 24 h after treatment with Bic, although not to the extent seen for Mib, and stayed constant for the remaining duration of the experiment (Figure 2B). Contrary to what was observed for Bic, addition of 17‐AAG (which inhibits HSP90 by blocking its interaction with p23) decreased AR to levels below those obtained for vehicle‐treated cells, but did not significantly affect endogenous p23 levels, and increased HSP90 levels 72 h after treatment (Figure 3B). Increased HSP90 levels following treatment with an HSP90 inhibitor has previously been reported, and attributed to cellular stress response following HSP90 inhibition (Lamoureux et al., 2011).

Results shown demonstrate p23 can affect AR protein levels, further confirming its role in the signalling pathway. Given the effects of p23 upon AR activity (Reebye et al., 2012) and stability (and vice‐versa) and since prostate cancer is an AR‐driven malignancy, we next investigated whether p23 may also be involved in promoting prostate cancer progression.

### 3.3 Altering p23 levels does not affect prostate cancer cell growth

**Figure 4:**
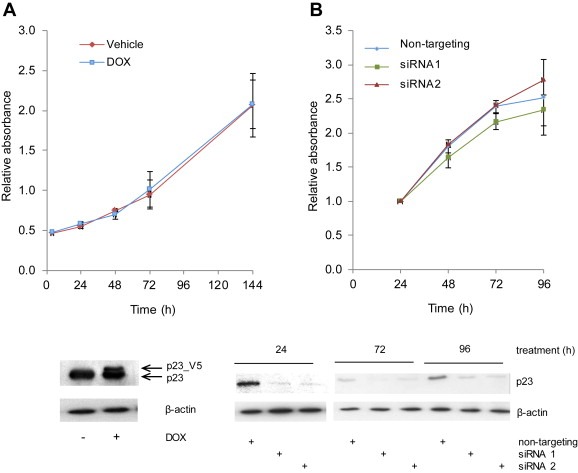
p23 levels do not affect cell growth. (A) LNCaP_p23 cells were treated with 100 nM doxycycline (DOX) for 48 h prior to seeding in 96‐well plates for cell viability assessment. Twenty microlitres of MTT reagent was added to each well, incubated for 2 h at 37 °C and absorbance measured at 490 nm at the specified time points. To verify p23 ectopic expression, cells were treated as above and lysates collected, harvested and resolved by Western blotting. Gels were transferred onto nitrocellulose membranes and probed with specific antibodies against p23 and β‐actin. (B) LNCaP cells were transfected with individual siRNAs against p23 or a scrambled control for 72 h prior to seeding in 96‐well plates for cell viability assessment, performed as in (A). To verify p23 knockdown cells were transfected and collected at the indicated time points. Lysates were resolved by SDS‐PAGE, transferred onto nitrocellulose membranes and probed with specific antibodies against p23 and β‐actin. Results are the mean ± 1 STDEV of two independent experiments performed in triplicate.

To elucidate whether p23 has a role in cell viability, growth assays were performed using the LNCaP‐p23 inducible line for enhanced p23 ectopic expression and parental LNCaP cells for endogenous p23 knockdown experiments. p23 ectopic expression and knockdown were verified by Western blotting (Figure 4A and B respectively). As shown in Figure 4A, over‐expression of p23 (which in the presence of doxycycline was increased by around 60%) did not affect cell growth at any of the time points analysed and no differences were observed when compared to vehicle‐treated cells. Concordantly, the converse experiment in which p23 was transiently knocked‐down also indicated no changes in cell viability when compared to the non‐targeting transfected control cells (Figure 4B). The results suggest p23 has no direct role in cell proliferation.

### 3.4 p23 increases prostate cancer cell motility and invasiveness

**Figure 5:**
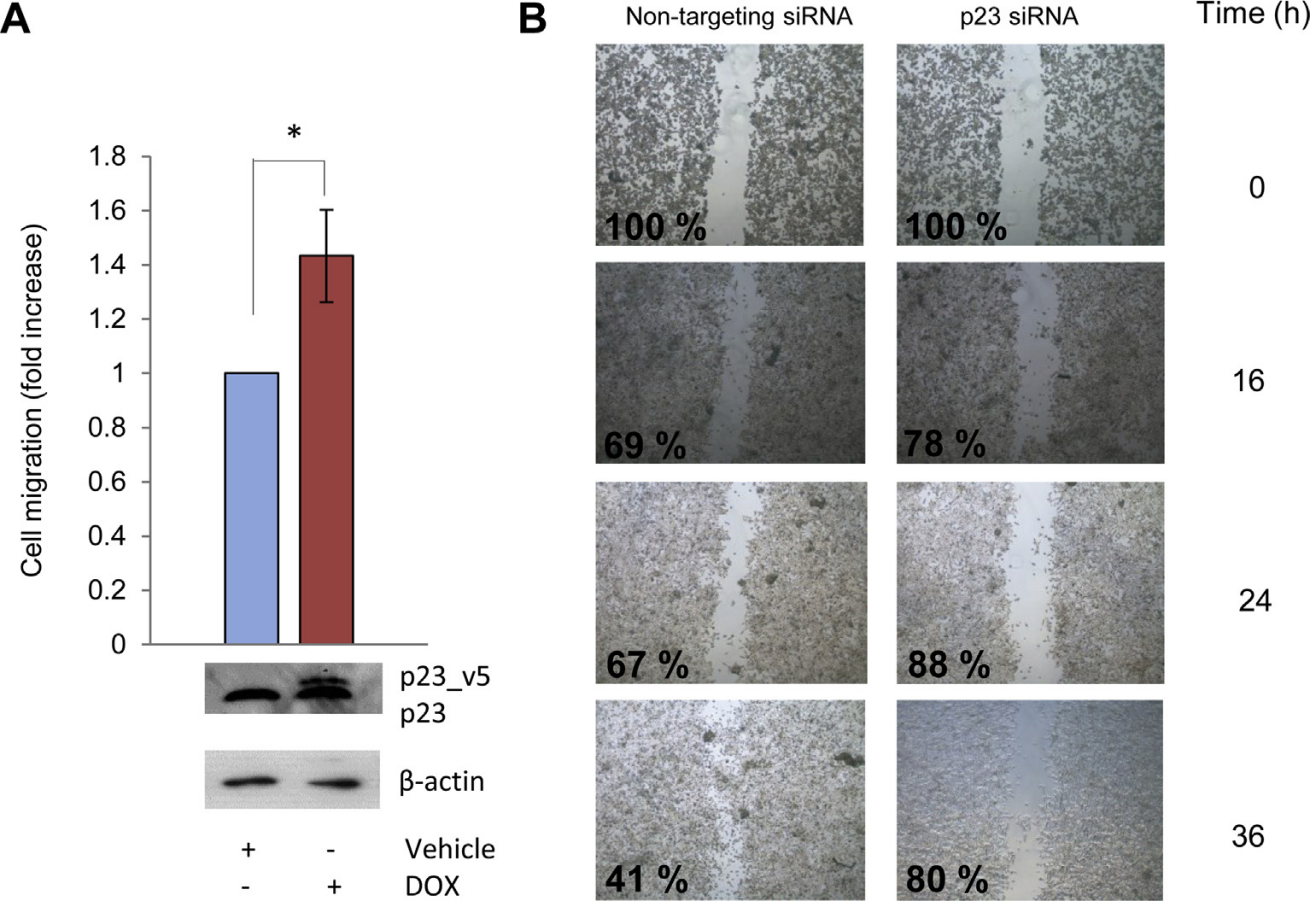
p23 levels affect cell migration. (A) LNCaP_p23 cells were treated with 100 nM doxycycline (DOX) for 48 h prior to seeding at a density of 1.5 × 10^4^ in serum free medium in the upper chamber of transwells pre‐coated with collagen for 2 h. Full media was placed in the lower chamber and used as a chemoattractant. After 24 h, cells in the lower chamber were fixed, stained and migration quantified using ImageJ. To verify p23 ectopic expression cells were harvested and lysates resolved by Western Blotting and probed with specific antibodies against p23 and β‐actin. Results are the mean ± 1 STDEV of a representative experiment performed in triplicate. (B) LNCaP cells were transfected with siRNA against p23 or non‐targeting control. Seventy‐two hours after cells were seeded to confluency in a 6‐well plate and 24 h after monolayers were scratched with a pipette tip, washed with PBS twice and incubated in hormone‐depleted media for further 36 h. Recovery of the wound was monitored and images were taken at the specified time points after scratching. The wound area was measured for each time point and condition using ImageJ and values were made relative to the corresponding wound at 0 h and expressed as a percentage. C4‐2.

The effects of p23 in metastasis formation and cancer progression have been previously studied in breast cancer but the role of p23 in prostate cancer spread is relatively unexplored (Oxelmark et al., 2006, 2012, 2010). We used transwell assays wherein an FCS gradient was created by adding full medium into the lower chamber and cells allowed to migrate for 24 h. Increased, ectopic expression of p23 significantly increased LNCaP‐p23 cell migration (Figure 5A) through the FCS gradient. Next, wound‐healing assays were performed in LNCaP cells transfected with either siRNA against p23 or a non‐targeting control (Figure 5B). Cells were incubated under starvation conditions to prevent cell proliferation, the monolayer scratched at maximal cell confluency and wound recovery monitored up to 36 h after wounding. The same field was imaged and wound area quantified for each condition throughout the time course. Both monolayers initially presented a very similar degree of confluency and both scratches were of similar length and width. Although progressive wound closure was observed in control cells, which colonised up to 60% of the initial wound area after 36 h, depleting endogenous p23 caused a delay in wound recovery with a maximal recovery of approximately 20%. Together, this data supports a role for p23 in cell motility and migration. We also performed wound healing assays in PC3 cells, which do not express functional AR, and found that depletion of p23 by siRNA also significantly reduced wound recovery i.e. cell motility (Supplemental Figure 1). This demonstrates that the effects of p23 on cell motility are mediated via non‐AR client proteins, although does not rule out involvement of AR in AR‐positive cells.

**Figure 6:**
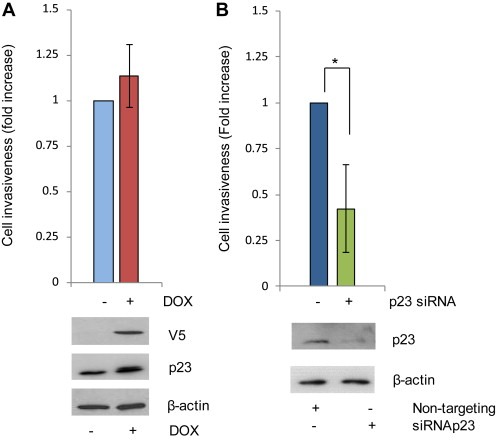
p23 increases cell motility. (A) LNCaP_p23 cells were treated as per Figure 5 and (B) LNCaP cells were transfected with siRNA against p23 or a scrambled control for 72 h prior to being seeded at a density of 1.5 × 104 in serum free medium in the upper chamber of transwells pre‐coated with collagen for 2 h and matrigel for further 2 h. Full media was placed in the lower chamber and used as a chemoattractant. After 24 h, cells in the lower chamber were fixed, stained and invasion quantified using ImageJ. To verify p23 overexpression (A) and knockdown (B) cells were harvested 72 h after transfection and lysates resolved by Western Blotting and probed with specific antibodies as indicated. Results are the mean ± 1 STDEV of a representative experiment performed in triplicate *p value ≤0.05 (Student's t test).

Since manipulating p23 levels affected cell motility/migration, we next investigated the effects on cell invasive potential using LNCaP‐p23 and parental LNCaP cells. Cells were treated as for migration assays but the transwell filter was coated with a matrigel layer prior to cell seeding. As shown in Figure 6A, and despite what was previously described for cell migration assays, p23 over‐expression did not significantly affect cell invasion, although a small increase in the number of invading cells was observed upon DOX treatment (Figure 6A). However, a strong decrease in cell invasion (2.5‐fold) was seen in cells transfected with siRNA against p23 compared to non‐targeting ‐transfected cells (Figure 6B). This supports a role for endogenous p23 in invasive potential of prostate cancer cells.

### 3.5 High p23 expression correlates with decreased survival and bone metastasis in prostate cancer patients

**Figure 7:**
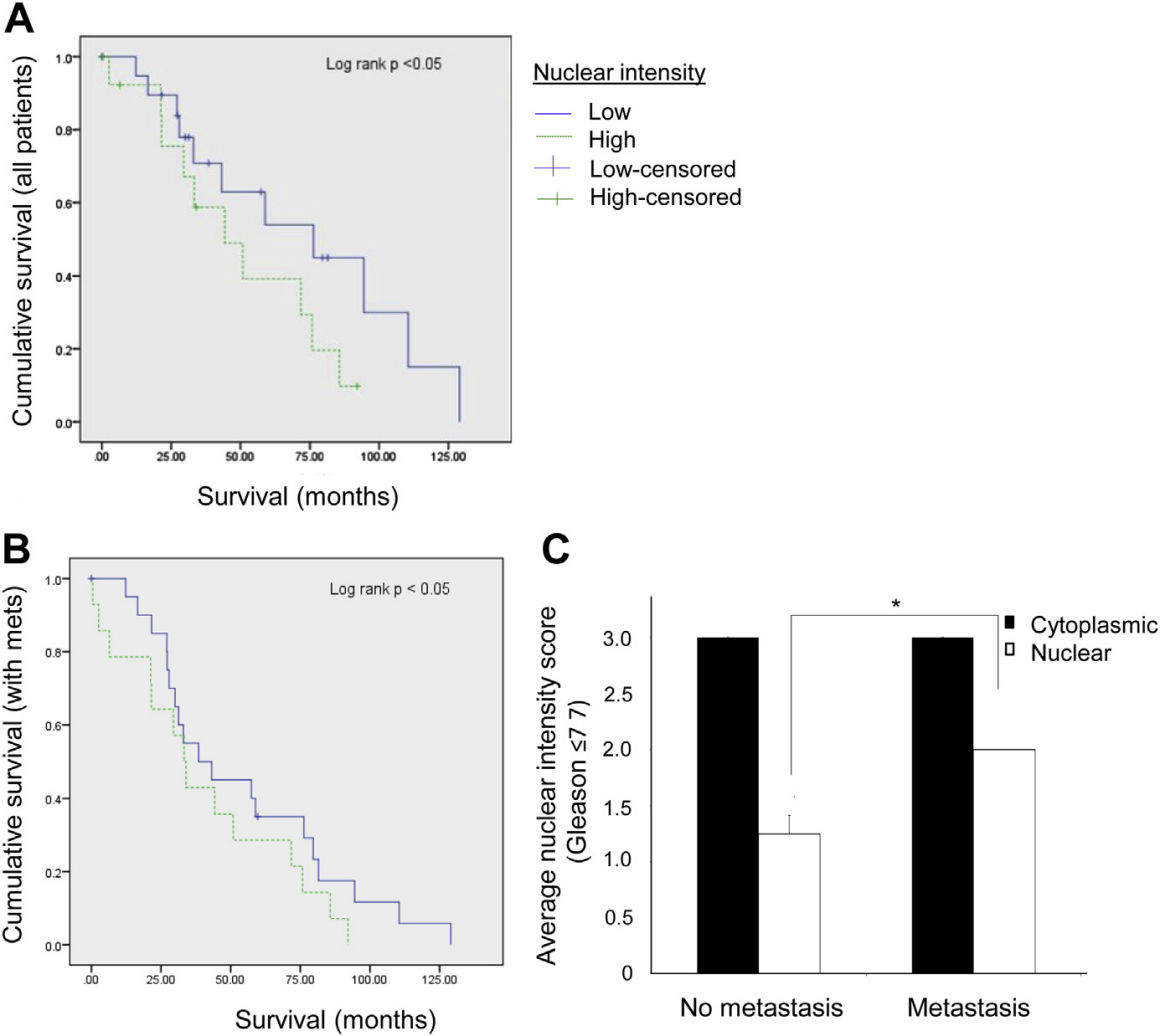
p23 expression directly correlates with patient survival and disease progression. Immunohistochemistry was performed on human prostate cancer tissue microarrays and cores scored for intensity of nuclear and cytoplasmic p23 staining. (A) Kaplan Meier graph depicting relation between overall survival and p23 nuclear staining in prostate cancer patients (n = 53). Low = score 0 or 1, High = score 2 or 3. (B) Kaplan Meier graph depicting relation between overall survival and p23 nuclear staining in prostate cancer patients with confirmed metastatic disease (n = 31). (C) Correlation between the intensity of nuclear and cytoplasmic p23 expression with the development of metastatic lesions in prostate cancer patients with Gleason score ≤7. *p value ≤0.05 (one‐tailed Student's t test).

As prostate cancer cells expressing higher p23 appeared to have increased invasive potential in our assays, we hypothesized that patients expressing high levels of p23 protein may be more prone to developing metastatic lesions and consequently have shorter survival times. To investigate this, immunohistochemistry was performed on prostate cancer tissue microarrays and specimens scored for primary Gleason grade and for nuclear and cytoplasmic p23 staining on a scale of 0–3 (3 = highest intensity). An inverse correlation between nuclear p23 expression and patient survival was observed. Patients with high nuclear p23 expression showed significantly shorter overall survival, with mean survival decreasing from 67 to 40.2 months and median from 57.4 to 31.7 months (Figure 7A and Table 1, *p* value = 0.017). Disease‐specific survival was also decreased in patients with high nuclear p23, with mean survival decreasing from 90.2 to 52.3 months (Supplemental Figure 2A and Supplemental Table S2). The survival differences were more significant in patients with lower grade tumours (Gleason grade ≤7, data not shown). Even in patients with confirmed metastatic lesions, there was a significant difference in survival between patients with high and low p23 (Figure 7B); reduction in survival was significantly correlated with high p23 nuclear expression suggesting p23 could serve as a poor prognostic indicator.

**Table 1:** Correlation of p23 staining with overall survival in prostate cancer patients. The intensity of nuclear p23 staining was correlated with patient data and median and mean overall survival times in prostate cancer patients were calculated.

Nuclear intensity	Overall survival time (months)							
	Meana				Median			
	Estimate	SE	95% CI		Estimate	SE	95% CI	
			Lower bound	Upper bound			Lower bound	Upper bound
Low	66.968	7.604	52.064	81.873	57.400	18.791	20.569	94.231
High	40.205	6.421	27.619	52.791	31.700	2.768	26.274	37.126
Overall	56.600	5.599	45.625	67.575	43.200	9.583	24.417	61.983

a Estimation is limited to the largest survival time if it is censored.

In agreement with our *in vitro* data, a direct correlation between the development of metastatic lesions and p23 nuclear levels was observed although this was only significant in patients with Gleason score ≤7 (Figure 7C). No significant differences were observed when analysing p23 cytoplasmic staining, further highlighting a role for nuclear p23 in prostate cancer progression.

### 3.6 Increased p23 levels cause changes in gene expression associated with invasive malignancies

**Figure 8:**
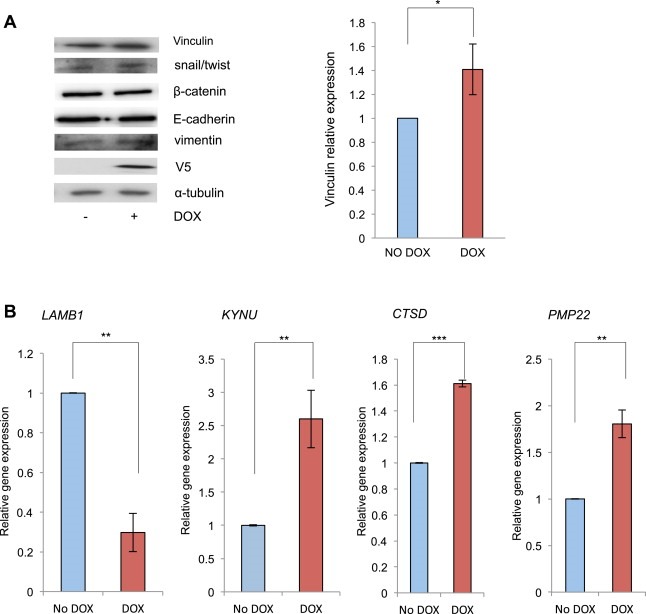
p23 specifically alters the expression of EMT components and re‐programmes chromatin transcription towards a more metastatic profile. (A) p23‐V5 tagged ectopic expression was induced in LNCaP‐p23 cells as explained before prior to cells being harvested and 10 μg of total lysate resolved by SDS‐PAGE, transferred onto nitrocellulose membranes and probed with specific antibodies against vinculin, V5 and α‐tubulin. Signal was quantified using ImageJ and normalised against the corresponding loading control. Results are the mean of 3 independent experiments. (B) LNCaP_p23 cells were incubated with or without DOX for 10 days prior to being harvested for RNA extraction. 500 ng of RNA were reverse‐transcribed and qRT‐PCR performed using specific primers against LAMB1, KYNU, CTSD and PMP22 genes. GAPDH was used as the reference gene. Results are the mean ± 1 STDEV of three independent experiments performed in triplicate. *p value ≤0.05 and **p value ≤0.005 (Student's t test).

To unravel the mechanism by which p23 affects cell motility, the protein expression levels of a panel of epithelial‐to‐mesenchymal transition (EMT) markers (snail, E‐cadherin, vimentin, β–catenin) was analysed in the inducible LNCaP‐p23 line. No changes in any of the markers analysed were observed despite increased V5‐p23 expression, suggesting p23 does not exert its effects upon cell motility in this manner (Figure 8A). However vinculin, a component of focal adhesions, was significantly increased (1.4 fold) in cells ectopically expressing p23, supporting previous observations that knocking down p23 reduces vinculin levels (Echtenkamp et al., 2011) and suggesting p23 may alter cell motility via modulating the interaction between the plasma membrane and the actin cytoskeleton.

Previously Simpson et al. demonstrated that, in breast cancer cells, p23 modulates the expression of a set of genes dysregulated in advanced breast cancer including those involved in migration, invasion, metabolism or transcriptional regulation (Simpson et al., 2012, 2010). To verify whether a similar mechanism could be responsible for p23 effects upon prostate cancer cell motility, the expression of the top p23‐regulated genes observed in the microarray study was analysed in our LNCaP‐p23 cells. We found that DOX treatment, i.e. increased p23 expression, significantly altered the expression of several such genes (Figure 8B) suggesting p23 may act by reprogramming gene expression profiles into more aggressive phenotypes. *KYNU, CTSD* and *PMP22* are known to be up‐regulated in advanced metastatic cancers and correlate with poor prognosis whereas *LAMB1* encodes a laminin‐1 protein shown to have reduced expression in breast cancer (Minn et al., 2005; Pruitt et al., 2013; Simpson et al., 2010; Tong et al., 2010).

## 4 Discussion

p23 is a molecular chaperone best characterised as an HSP90 co‐chaperone responsible for inhibiting the ATPase activity of the HSP. Less is known regarding other roles for p23, although it is important in several cellular processes, including modulating the activity of SRs such as AR, ER, GR and PR (Freeman and Yamamoto, 2002; Knoblauch and Garabedian, 1999; Reebye et al., 2012). We previously demonstrated that p23 enhances AR transcriptional activity and DNA‐binding. We now describe that p23 appears to be also important in stabilising AR in the absence of hormone since modulating levels of p23 protein resulted in changes in AR levels. Ectopically enhanced expression of p23 resulted in increased AR levels in the absence of hormone, which could be due to stabilisation/prevention of degradation of unliganded AR in the cytoplasm. Conversely, transient p23 knockdown significantly increased AR protein turnover. We have thus demonstrated the prominent role of p23 in foldosome complex function, as altering p23 levels alone without affecting the major component of this complex, HSP90, is sufficient to significantly affect AR. Importantly, this occurred in the absence of androgens, which mimics the situation in prostate cancer patients undergoing androgen deprivation therapy. The mechanism by which this occurs remains to be elucidated although it is likely to involve the previously demonstrated direct interaction between AR and p23 (Reebye et al., 2012), or could be via modulation of the HSP90 ATPase activity, which would in turn result in a stabilisation of the mature form of the SR heterocomplex. It is unlikely that these effects occur at the genomic level given that p23 has not been described to bind any regulatory regions in the vicinity of the *AR* gene (Massie and Mills, 2011; Sharma et al., 2013) and addition of Mib did not significantly alter p23 gene expression (Supplementary Figure S2). A similar mechanism has been described for HSP27, knockdown of which has been shown to promote AR degradation through the proteasome pathway while AR mRNA remained unchanged (Zoubeidi et al., 2007).

Although metastasis (usually to the bone) is the most lethal development of prostate cancer, very little is known about the mechanisms involved. We have provided evidence that p23 may be implicated in driving prostate cancer progression and metastasis although no effects on prostate cancer cell growth/viability were observed upon manipulation of p23 levels. Our results support previous reports by Oxelmark et al. which showed, in a breast cancer model, that p23 over‐expression neither affected MCF‐7 cell growth nor enhanced the expression of growth‐related genes, despite increasing transcription of other ER target genes (Oxelmark et al., 2006). In our studies, p23 over‐expression significantly enhanced cell migration while p23 knockdown decreased cell motility and invasiveness potential. While increasing p23 levels did increase invasiveness this was not significant, suggesting that p23 is required but not limiting for this process. p23 has been already shown to enhance MCF‐7 cell invasion and adhesion, thus potentially facilitating tumour invasion in breast, and our findings confirm a role for p23 as a regulator of events associated with these processes in prostate cancer cells (Oxelmark et al., 2006; Simpson et al., 2010). Further supporting this possible role of p23, ectopic expression of the chaperone enhances vinculin expression, which is implicated in the epithelial to mesenchymal transition (EMT) and tumour spreading. Conversely, p23^−/−^ MEFs present a vinculin impairment (Echtenkamp et al., 2011), strongly suggesting involvement of p23 in driving tumour cells towards high invasion profiles via modulating some EMT‐dependent cellular changes and interactions within cytoskeleton components. HSP90 has also been implicated in cell motility and metastatic processes since HSP90 over‐expression enhanced wound recovery (Tsutsumi and Neckers, 2007). Moreover, HSP90 also promotes activation of extra‐cellular proteins such as MMP‐2, leading to enhanced tumour invasiveness (Eustace et al., 2004; Stellas et al., 2010). Given the interactions between p23 and HSP90 and the fact that p23 as well as HSP90 has been found in conditioned media of fibrosarcoma cells (Eustace and Jay, 2004), it is plausible to infer that p23 is also indirectly involved in these processes through its role as an HSP90 co‐chaperone. Concomitantly, immunostaining performed on prostate cancer biopsies showed an association between eventual development of metastatic lesions with high nuclear p23 expression in tumours graded Gleason ≤7 at diagnosis. This data indicates that p23 could serve as a prognostic indicator, to assess the likeliness of developing metastatic lesions in patients with low Gleason stage tumours. There is a clinical need to stratify such patients for metastatic risk as early as possible, to target aggressive treatment to patients who will benefit and avoid overtreatment of others. Moreover, and further confirming a role for p23 in driving metastatic processes, we have reported that over‐expression of p23 is sufficient to cause some reprogramming of gene transcription into a potentially more aggressive phenotype. This is in agreement with previous findings reported in breast cancer cells, suggesting that the pathway by which p23 acts upon gene expression is conserved across different tissues and cell types, and is not dependent upon AR (Echtenkamp et al., 2011; Simpson et al., 2010).

The chaperoning complex is a potentially important therapeutic target in advanced prostate cancer, given the significance of HSP client proteins including growth factors and the AR (Ischia et al., 2013). Reducing p23 levels impacted upon cell proliferation, motility and migration, and this included reducing motility of AR‐negative cells. This is consistent with the effects being mediated through other HSP90 client proteins or co‐chaperones but does not rule out some contribution of AR, which has previously been described by other groups (Castoria et al., 2011, 2003, 2008). Given that we have previously demonstrated a p23 interaction with AR, and that nuclear translocation and enhancement of AR activity are partially HSP90‐independent, we postulate that selectively inhibiting p23 in combination with HSP90 could be a more efficient therapeutic strategy than HSP90 inhibition alone. This is supported by the fact that analysis of prostate cancer tissue microarrays highlighted a correlation between high nuclear levels of p23 and shorter overall and disease‐specific survival times. In further support of this, analysis of the transcriptomic profiles of a publically available dataset (Taylor et al., 2010) of prostate cancer (http://www.ncbi.nlm.nih.gov/geo/query/acc.cgi?acc=GSE21034) shows high p23 expression in prostate tumours correlated with decreased overall survival (Supplementary Figure S2B).

In conclusion, we have provided evidence supporting a role of p23 and HSP90 in AR signalling and disease progression involving the acquisition of increased cell motility and invasiveness potential, which appears to translate to the clinical scenario whereby high nuclear p23 is associated with and may drive increased occurrence of metastasis. Data presented here suggests HSP90 and p23 are involved in driving these cellular processes, further supporting these proteins as valid therapeutic targets in prostate cancer, and in the case of p23 as a putative prognostic indicator for metastatic progression in patients with lower grade tumours at diagnosis, an area of unmet clinical need.

## 5 Conflict of interests

The authors declare no conflict of interests.

## Supporting information



The following is the supplementary data related to this article:
Supplementary Information accompanies the paper.

Supplementary data

## Footnotes

^3^ Non‐standard abbreviations: AR, androgen receptor; ER, oestrogen receptor; HSP90, heat shock protein 90; EMT, epithelial to mesenchymal transition; DOX, doxycycline; Mib, mibolerone.

## Supplementary data 1

### 1.1

Supplementary data related to this article can be found at http://dx.doi.org/10.1016/j.molonc.2014.08.014.
