# Supplementary material for: The co‐chaperone p23 promotes prostate cancer motility and metastasis
Source: Mol Oncol. 2014 Sep 6;9(1):295–308. doi: 10.1016/j.molonc.2014.08.014 (PMC4510206; doi:10.1016/j.molonc.2014.08.014)
Supplement: Supplementary file 1 — Supplementary data [file MOL2-9-295-s001.pdf]

Querol Cano et al - Supplemental Data

LAMB1	5'-AGGAACCCGAGTTCAGCTAC-3'<br>5'-CACGTCGAGGTCACCGAAAG-3'
KYNU	5'-GGCTCTCCACCTAGATGAGGA-3'<br>5'-GCTGCTATTTTGGCCCACTTAT-3'
Cathepsin D	5'-GTACATGATCCCCTGTGAGAAGGT-3'<br>5'-GGGACAGCTTGTAGCCTTTGC-3'
PMP22	5'-GTGCTGCTGTTCGTCTCCAC-3'<br>5'-ATCAGTTGCGTGTCCATTGC-3'
GAPDH	5'-ATGGGGAAGGTGAAGGTCG-3'<br>5'-GGGGTCATTGATGGCAACAATA-3'

Table S1 qRT-PCR Gene expression primers

	Disease-specific Survival Time (Months)							
Nuclear p23 intensity	Mean[a]				Median			
	Estimate	SE	95% CI		Estimate	SE	95% CI	
			Lower Bound	Upper Bound			Lower Bound	Upper Bound
Low	90.165	9.722	71.110	109.220	109.200	19.720	70.548	147.852
High	52.317	8.470	35.716	68.918	44.300	10.683	23.361	65.239
Overall	76.528	7.593	61.646	91.409	75.800	17.854	40.806	110.794

a. Estimation is limited to the largest survival time if it is censored.

Table S2 Correlation of p23 staining with the disease-specific survival time in prostate cancer patients. The intensity of nuclear p23 staining was correlated with patient data and median and mean overall survival times in prostate cancer patients were calculated.

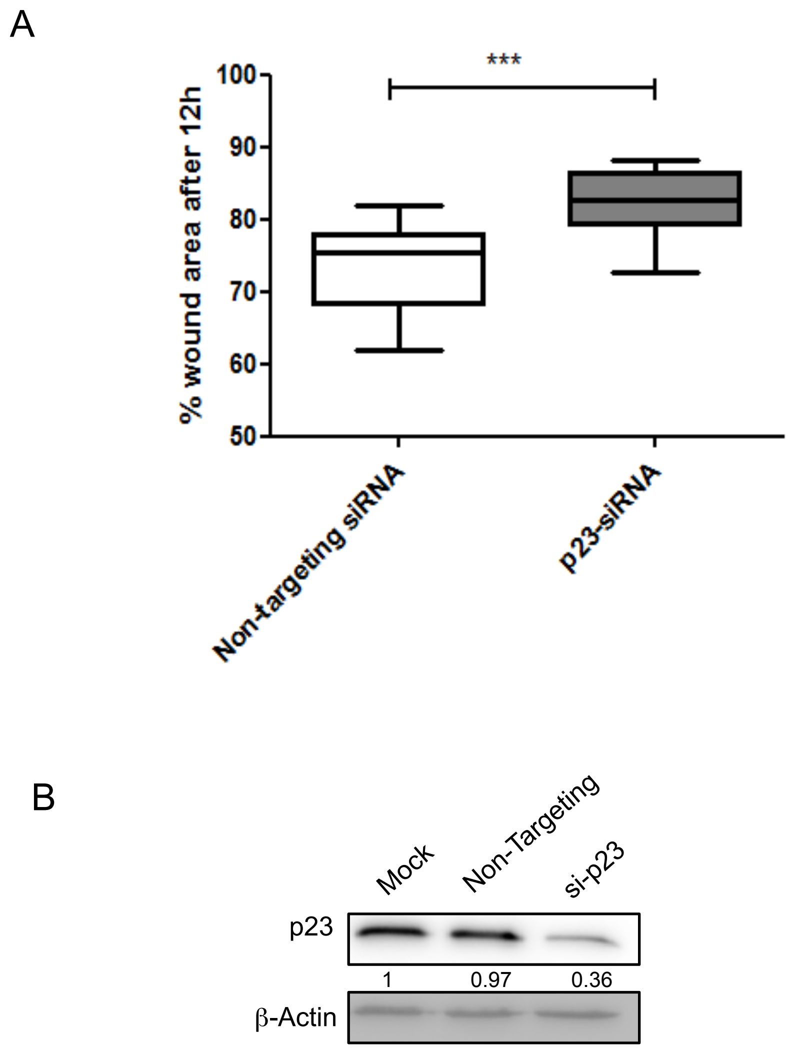

Figure S1 (**A)** PC3 cells were transiently transfected with siRNA specific for p23 or non-targeting control. 72 h later the cell monolayer was scratched using a pipette tip, washed with PBS and placed in hormone-depleted media. Cell migration was imaged every 30 min for 12 h. Data shown are of 2 independent experiments performed in duplicate and represent migration after 12h. The wound area was measured for each time point and condition using ImageJ and values are expressed as percentage wound area remaining, relative to the corresponding wound at 0h set at 100%. A Mann Whitney test was used to determine the significance of the difference in cell migration compared to the corresponding control (*** $p < 0.001$). (**B)** Immunoblot analysis 72 h after siRNA transfection to confirm p23 knockdown. Relative amount of p23 protein is given under p23 blot (densitometry of p23 blot normalised for ß-actin).

Querol Cano et al - Supplemental Data

A

B

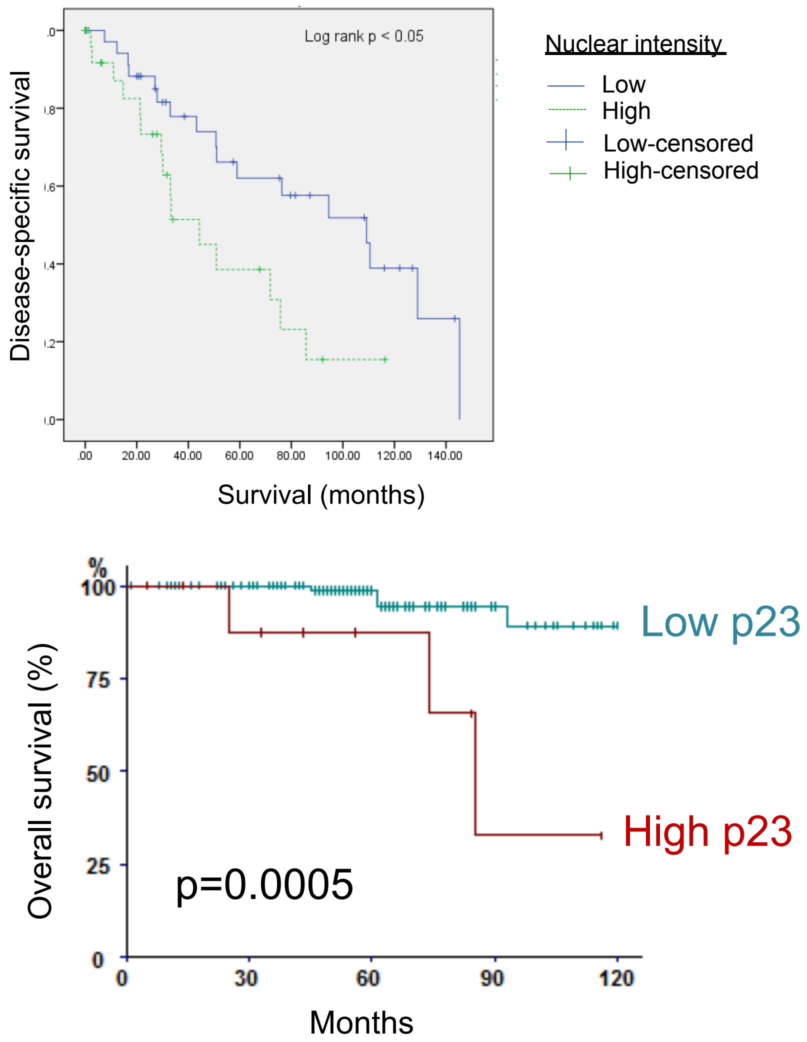

Figure S2 (A) Correlation of p23 staining with disease-speciific survival in prostate cancer patients - Kaplan Meier graph depicting relation between disease specific survival functions and p23 nuclear staining in prostate cancer patients with confirmed metastatic disease. **(B)** Analysis of overall survival over time for patients with tumours with high p23 expression levels (red line) and low p23 expression levels (blue line). Data is taken from Ref. 57. The optimal cutoff point to categorize prostate cancer patients into low – and high –p23 expression was evaluated by use of the receiver operating characteristic method. To determine whether p23 expression is associated with the patient clinical outcome, survival distributions were estimated by the Kaplan-Meier method, and the significance of differences between survival rates was ascertained using the log-rank test.

Querol Cano et al - Supplemental Data

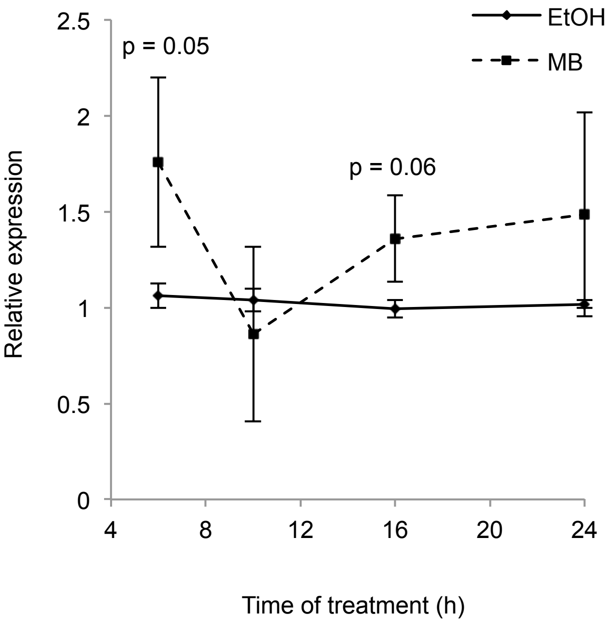

Figure S3 LNCaP cells were depleted for hormone for 72 h and treated with 10 nM Mibolerone (Mib) for 6, 10, 16 and 24 h. Cells were collected, mRNA extracted and the resulting cDNA analysed for p23 expression with qRT-PCR. Data shown represents the mean ± 1 STDEV of three independent experiments performed in duplicate.
